# Supplementary material for: Scanning electron microscopy preparation of the cellular actin cortex: A quantitative comparison between critical point drying and hexamethyldisilazane drying
Source: PLoS One. 2021 Jul 7;16(7):e0254165. doi: 10.1371/journal.pone.0254165 (PMC8263306; doi:10.1371/journal.pone.0254165)
Supplement: Attachment — Submitted filename: Response to Reviewers.docx [file pone.0254165.s001.docx]

**Comments to the Author**

**Please note: All comments and answers written by the authors are marked in red.**

1. Is the manuscript technically sound, and do the data support the conclusions?

The manuscript must describe a technically sound piece of scientific research with data that supports the conclusions. Experiments must have been conducted rigorously, with appropriate controls, replication, and sample sizes. The conclusions must be drawn appropriately based on the data presented.

Reviewer #1: Yes

Reviewer #2: Yes

Reviewer #3: Partly

2. Has the statistical analysis been performed appropriately and rigorously?

Reviewer #1: Yes

Reviewer #2: Yes

Reviewer #3: No - We hope the additional ANOVA analysis is going to fulfill this question now.

3. Have the authors made all data underlying the findings in their manuscript fully available?

The [PLOS Data policy](http://www.plosone.org/static/policies.action#sharing) requires authors to make all data underlying the findings described in their manuscript fully available without restriction, with rare exception (please refer to the Data Availability Statement in the manuscript PDF file). The data should be provided as part of the manuscript or its supporting information, or deposited to a public repository. For example, in addition to summary statistics, the data points behind means, medians and variance measures should be available. If there are restrictions on publicly sharing data—e.g. participant privacy or use of data from a third party—those must be specified.

Reviewer #1: Yes

Reviewer #2: Yes

Reviewer #3: No - We thought according to our understanding of the PlosOne definition that the presentation of data points within the figures are sufficient. Hence, we are not fully sure what you are referring to. If needed, we can provide the raw data (e.g. in table-format) upon request any time.

4. Is the manuscript presented in an intelligible fashion and written in standard English?

PLOS ONE does not copyedit accepted manuscripts, so the language in submitted articles must be clear, correct, and unambiguous. Any typographical or grammatical errors should be corrected at revision, so please note any specific errors here.

Reviewer #1: Yes

Reviewer #2: Yes

Reviewer #3: Yes

5. Review Comments to the Author

Please use the space provided to explain your answers to the questions above. You may also include additional comments for the author, including concerns about dual publication, research ethics, or publication ethics. (Please upload your review as an attachment if it exceeds 20,000 characters)

Reviewer #1: The authors present a comparative study about different preparation (drying) methods of brain cortex for scanning electron microscopy. They compare standard criyical point drying (CPD), with CDP plus lens tissue (CDP-LT), and hexamethyldisilazane drying, of which the last method created the least number of artifacts (hole, etc.). Effects of the methods on cell thickness have been monitored by atomic force microscopy. This is a concise paper, and all the results seem flawless and appropriately executed. This may be a relevant study for all these locations that cannot resort to any cryo methods, and where the low-resolution results shown here still bear enough data for a scientific comparison or analysis.
The issue I have with this paper is more of a philosophical nature, than a technical one. The gold-standard for preparations today of cells and cellular components should always be cryo-SEM (or cryo-EM in general), even if this is not used, e.g., in a high-throughput approach, or in a clinical environment, where results have to be achieved rapidly. Cryo-EM or cryo-SEM warrants the least interference with distortions, and as such, all other methods should be compared to this. In this study, all methods used, no matter how standardized and commonly used they are, produce large amounts of distortions and alterations to the cellular fine structures. Hence, I would consider cryo-methods the gold standard, to which everything else should be compared to (especially for the atomic-force height measurements). That will not reduce the relevance of the techniques tested here, but it would place them into a more realistic perspective of what all this drying is actually doing to a biological sample, no matter which method is chosen.
To make this paper acceptable for me, I would like to see a comparison to cryo-preparations. There are serious artifacts from all three preparations that render an objective comparison quite difficult. I believe the results shown here, but I am not sure that one method (HMDS) is truly superior and should allow more sophisticated conclusions than the other two. Hence, I recommend acceptance after major revisions (adding cryo-data). Cryo-SEM is quite common these days, especially in Germany. There are multiple institutions that could help with that.

Thank you very much for your effort of pointing out the outstanding benefits of Cryo-SEM. We do fully agree with you that Cryo-SEM in combination with Cryo-Tomography is an outstanding technique, which can provide even 3D reconstructions of the actin cortex as is was excellently summarized by Chakraborty and co-worker in 2020 (Chakraborty, Jasnin and Baumeister “Three dimensional organization of the cytoskeleton: a cryo-electron tomography perspective”; DOI: 10.1002/pro.3858). Since we are in contact with a few groups working on Cryo-EM in context of the cellular actin cortex, we know that this technique is extremely challenging, time consuming and very costly. Moreover and even more relevant, it is typically performed on thin actin cortex layers of adhered cells (e.g. lamellipodia), but not on cellular regions like the perinucleus or above the nucleus. We totally agree with you that this could be possible, although technically extremely challenging (correct and precise orientation of detached adhered cells embedded in a tomography suitable environment plus identification of the target region within the images of such “slices” cell regions). This might be the reason why a broad range of actin-cortex groups (e.g. Ewa Paluch, Guillaume Charras, Tatyana Svitkina etc.) are using techniques similar to the ones we are presenting in our manuscript. Since these drying-techniques are actively used up to today and (in our eyes) more frequently than Cryo-EM (if at all in the cell-region depending context), we decided to optimize the standard CPD LT with the use of hmds as described in the manuscript. Moreover, the method we are using is significantly cheaper than Cryo-EM, which potentially enables groups all over the world to make use of it and gain high-resolution insights of the cellular actin cortex structure; even if they do not have access to financial funding required for cryo EM. In our eyes, this is an outstanding advantage of our method.

Nonetheless, we performed both Cryo-fracture and transmission electron microscopy on suspended cells (since they are technically manageable compared to adhered cells presented in our study). However, we actively decided against using Cryo-EM or TEM since Cryo-fracture is far away from the targeted resolution (contrasting only possible under very specific conditions) and transmission electron microscopy is not useful without the ability of tomography graphs. For a better impression please see the figure that we added here.

In summary, we totally agree with you that Cryo-EM tomography is the golden way, but up to today still far away from being standard since the complexity of preparation, needed equipment and total costs are very high. In contrast, and to our best knowledge, we are the first group successfully using hmds instead of CPD LT for the drying preparation of the cellular actin cortex for SEM (and for all cell regions of interest) and found that the cellular height is significantly better conserved for hmds than for CPD LT. Again, we do agree with you that artifacts are present for all investigated drying methods, while we do not expect AMH or significant height shrinkage upon using Cryo-EM. Nonetheless, a direct comparison of Cryo-EM to the presented methods is very challenging since A) AMH are almost impossible to identify in Cryo-EM images and B) cells are typically embedded in specific substances before shock freezing, which changes the boundary conditions significantly. Therefore, investigating the cellular height would not be possible with AFM anymore. We targeted the issue of AMH with intensity thresholding as explained. Moreover, we performed several studies upon altering the cellular actin cortex by compounds and we can clearly identify significant differences in the structural parameters of the actin cortex using hmds drying (manuscript is currently in the writing process). Consequently, even if Cryo-EM tomography is obviously the superior method to investigate the actin cortex, we think that the methods we compared are absolutely state of the art and hence very useful, especially in combination with our free analyzing software FiNTA, which allows the direct intensity thresholding and therefore minimizing the analysis of artifacts. We hope that you agree on our argumentation and thank you again very much for this very important comment.

Reviewer #2: Manuscript is done, the authors clearly discuss the pro and cons of different drying methods for Electron microscopy.

I would like the authors to address the figure 4, Please include the magnification, scale bar on the SEM images. Thank you very much for this comment. We double-checked all scale bars and added them.

Reviewer #3: 1. In Fig 2B, please show the images for HMDS 1, HMDS25 and HMDS50 so that its easy to compare them. Please use Anova to test the significance as there are more than 2 groups. We thank you very much for providing this ideas. We think the representative images are very useful for the reader and clearly improve the manuscript as well as the consistent ANOVA anlysis.

We also performed ANOVA tests as suggested. All significance trends remain the same. We added the ANOVA significance information for all requested data sets within the manuscript (Figure captions). For this particular data set of Fig. 2B, the requested ANOVA p-value was 1.88005e-21.

2. Is Fig.2C quantification for Fig 1? Please clarify. This is correct and now updated. Thank you very much for this hint.

We also performed ANOVA tests on this data set. The p - value was 5.23583e-08.

3. Fig 3FA, B, D,E (now B, C, E, F) please show the representative fluorescence and SEM images. Please specify "n" number in the legend. Use Anova to test significance. Thank you very much for this well-spotted comment. We added the representative images as well as N within all Figures as well as the ANOVA tests. Obviously, these clearly improved the manuscript.

The requested ANOVA tests resulted in the following p-Values:

- 3B) p = 0.5785
- 3C) p = 5.97901e-11
- 3E) p = 5.42875e-30
- 3F) p = 4.9223e-24

4. In Fig 3, please show the atomic absorption images of living, fixed and CPD. Since the essential outcome of our manuscript is that either hmds or CPD LT are the methods of choice, we only performed full AFM-imaging on those two for illustration and re-emphasizing the essential difference between both. All other height measurements were performed by indenting the nuclear and perinuclear region only once per cell (respectively one pixel of the AFM-images). We agree that for completeness, the AFM images of living, fixed and CPD treated cells would be useful. Nonetheless, since our main focus is to point out the difference between hmds and CPD LT, we prefer to only show hmds and CPD LT AFM-images. We hope that this strategy is acceptable.

5. In Fig. 4, please show representative images for B, D, F. Please use anova to test the significance since comparison is between 3 groups. Thank you very much for this comment. Representative images are shown in Figure 1. Since the parameters MHA, connectivity and fiber length are the result of an analysis of several 10k MHA, we prefer to avoid “representative” images in this specific case (exemplary images would be potentially misleading). Nonetheless, we think it is very useful to support Fig. 4 A, C, E with examples for real images as we have added now. We hope that you can accept this.

As requested, we also performed the ANOVA tests for the data sets represented in Fig. 4 B, D, F. No significance trends changed. The particular p-values are:

- Fig 4B: p = 7.4258e-04
- Fig. 4 D: p = 1.42071e-05
- Fig. 4 F: p = 3.69289e-07
